# Triple-Gene Therapy for Stroke: A Proof-of-Concept *in Vivo* Study in Rats

**DOI:** 10.3389/fphar.2018.00111

**Published:** 2018-02-15

**Authors:** Mikhail E. Sokolov, Farid V. Bashirov, Vage A. Markosyan, Tatyana V. Povysheva, Filip O. Fadeev, Andrey A. Izmailov, Maxim S. Kuztetsov, Zufar Z. Safiullov, Maxim M. Shmarov, Boris S. Naroditskyi, András Palotás, Rustem R. Islamov

**Affiliations:** ^1^Department of Medical Biology and Genetics, Kazan State Medical University, Kazan, Russia; ^2^Gamaleya Research Institute of Epidemiology and Microbiology, Moscow, Russia; ^3^Institute of Fundamental Medicine and Biology, Kazan (Volga Region) Federal University, Kazan, Russia; ^4^Asklepios-Med (Private Medical Practice and Research Center), Szeged, Hungary; ^5^Kazan Institute of Biochemistry and Biophysics, Russian Academy of Sciences, Kazan, Russia

**Keywords:** adenoviral vector, GDNF, gene therapy, human umbilical cord blood mono-nuclear cell, middle cerebral artery, NCAM, stroke, VEGF

## Abstract

Natural brain repair after stroke is extremely limited, and current therapeutic options are even more scarce with no clinical break-through in sight. Despite restricted regeneration in the central nervous system, we have previously proved that human umbilical cord blood mono-nuclear cells (UCB-MC) transduced with adenoviral vectors carrying genes encoding vascular endothelial growth factor (VEGF), glial cell-derived neurotrophic factor (GDNF), and neural cell adhesion molecule (NCAM) successfully rescued neurons in amyotrophic lateral sclerosis and spinal cord injury. This proof-of-principle project was aimed at evaluating the beneficial effects of the same triple-gene approach in stroke. Rats subjected to distal occlusion of the middle cerebral artery were treated intrathecally with a combination of these genes either directly or using our cell-based (UCB-MC) approach. Various techniques and markers were employed to evaluate brain injury and subsequent recovery after treatment. Brain repair was most prominent when therapeutic genes were delivered via adenoviral vector- or UCB-MC-mediated approach. Remodeling of brain cortex in the stroke area was confirmed by reduction of infarct volume and attenuated neural cell death, depletion of astrocytes and microglial cells, and increase in the number of oligodendroglial cells and synaptic proteins expression. These results imply that intrathecal injection of genetically engineered UCB-MC over-expressing therapeutic molecules (VEGF, GDNF, and NCAM) following cerebral blood vessel occlusion might represent a novel avenue for future research into treating stroke.

## Introduction

A lack of available and effective treatments for ischemic injury results in the high mortality and disability of patients with stroke throughout the world. The current strategy to prevent brain cell death in the penumbra (around the ischemic core) proposes employment of stem cell therapies by direct (*in vivo*) or cell-mediated (*ex vivo*) gene therapy. The exponential growth of biotechnology methods in regenerative medicine for treatment of incurable and devastating diseases has resulted in some clinical successes. Clinical trials in gene therapy for central nervous system (CNS) disorders have been reported for treatment of Alzheimer’s (Rafii et al., 2014) and Parkinson’s (Warren Olanow et al., 2015) diseases. These clinical investigations failed to show therapeutic efficacy however proved the safety of the intracranial injection suggesting a potential route of recombinant viral vectors carrying therapeutic genes after stroke. There have been clinical trials employing autologous or allogeneic types of cells for patients with stroke (Jeong et al., 2014). The strategy for *ex vivo* gene therapy employing hematopoietic stem cells was successfully used for treatment of severe congenital immune deficiencies. For immunodeficiency disorders, CD34 positive cells harvested from the patient’s bone marrow or peripheral blood were transduced with an integrating viral vector expressing adenosine deaminase (Aiuti et al., 2017), WAS protein (Hacein-Bey Abina et al., 2015), or γc cytokine receptor (Cavazzana-Calvo et al., 2000) and reinfused into the same patient. Recently, to treat X-linked adreno-leuko-dystrophy (a fatal neurodegenerative disease caused by mutations of the ABCD1 gene encoding an adenosine triphosphate-binding cassette transporter localized in the membrane of peroxisomes in oligodendrocytes and microglia), the autologous CD34 positive cells infected with lentiviral vector encoding wild-type ABCD1 were reinfused to stop progressive cerebral demyelination in patients (Cartier et al., 2009).

In contrast the effectiveness of gene therapy for stroke is shown only in ischemic stroke animal models (Rhim and Lee, 2016). Local intracerebral or intraventricular injections of viral vectors carrying various genes encoding neurotrophic factors [BDNF, CNTF, glial cell-derived neurotrophic factor (GDNF), vascular endothelial growth factor (VEGF)], anti-apoptotic proteins (Bcl-2, Bcl-XL), heat shock proteins (Hsp25, Hsp70), and anti-inflammatory molecules (IL-1RA) have been evaluated for the treatment of ischemic stroke (Craig and Housley, 2016; Rhim and Lee, 2016). The success of cell therapy of stroke with neural precursors raised from embryonic stem cells (Drury-Stewart et al., 2013) and induced pluripotent stem cells (Mohamad et al., 2013; Yuan et al., 2013), mesenchymal stem cells (MSC) obtained from bone marrow (He et al., 2016), and umbilical cord blood (Lim et al., 2011; Zhu et al., 2015) encouraged the use of the same cell lines as gene delivery cellular carriers. Thus, MSCs were used for delivery of the genes encoding BDNF (Nomura et al., 2005), PIGF (Liu et al., 2006), and VEGF (Chen et al., 2015) to the ischemic brain. Cell-mediated gene delivery makes the viral antigens inside the *ex vivo* transduced cells invisible to the recipient immune system. Previously, we have shown that intravenous administration of UCB-MC transduced with three adenoviral vectors carrying VEGF, GDNF, and neural cell adhesion molecule (NCAM) improved symptomatic outcomes and increased life-span in transgenic ALS mice (Islamov et al., 2016), in rat (Islamov et al., 2017a), and mini-pigs (Islamov et al., 2017b) with spinal cord injury (SCI) model. The beneficial neuroprotective effects of the chosen therapeutic molecules are based on their biological role in the regulation of neurogenesis and neuroregeneration by promoting neuron survival and maintaining their morphological and physiological plasticity. VEGF is well known as a potent mitogen for vascular endothelial cells and is a typical neurotrophic factor. Thus, VEGF during stroke recovery can support microvascular angiogenesis and may have powerful neuroprotective and neurorestorative effects in the ischemic brain, shown in numerous preclinical studies (Zhang et al., 2000).

The main function of GDNF is the protection of neural cells. In the ischemic brains, GDNF promotes survival of neural cells and increases the number and facilitates migration of neuroblasts (Kobayashi et al., 2006). The role of cell adhesion molecules as targets of stroke therapy was addressed using leukocyte and platelet adhesion properties in post-ischemic cerebral microvasculature and inflammatory signaling pathways activation. Adhesion molecule NCAM is expressed on the surface of neurons and glial cells. During neurogenesis and neuroregeneration NCAM-mediated intercellular interactions provide for the survival and migration of neurons, axon growth cone guidance, and synaptogenesis (Colombo and Meldolesi, 2015). In the used recombinant NCAM in UCB-MC may facilitate cells migration to the ischemic area, increase their viability, and extend production of the therapeutic molecules by UCB-MC.

The current study is designed as a proof-of-principle to investigate the therapeutic efficacy of *in vivo* and *ex vivo* triple gene therapy (VEGF, GDNF, and NCAM) for increasing survivability of the affected neurons in a rat model of stroke.

## Materials and Methods

### Adenoviral Vectors

Viral vectors carrying human therapeutic genes *VEGF165, GDNF*, and *NCAM1* and reporter green fluorescent protein gene *GFP* were constructed based on the recombinant replication-defective adenovirus serotype 5 (Ad) in Gamaleya Research Institute of Epidemiology and Microbiology (Moscow, Russia) as described previously (Shcherbinin et al., 2014). The nucleotide sequences encoding VEGF165 (Gene Bank NM_001171626.1), GDNF (Gene Bank NM_019139.1), and NCAM1 (Gene Bank NM_001076682.2) were obtained by chemical synthesis in “Evrogen” JSC. Human and reporter genes were cloned into shuttle plasmid vectors pShuttle-CMV (Stratagene, La Jolla, CA, United States). Homologous recombination was then carried out for pShuttle-VEGF, pShuttle-GDNF, pShuttle-NCAM1, and pShuttle-GFP with pAd-Easy plasmids in *Escherichia coli* strain BJ5183 cells (Stratagene, La Jolla, CA, United States). Recombinant Ads were grown in HEK-293 cell culture and purified by exclusion chromatography. The titers of Ad-VEGF165 (2.2 × 10^9^ PFU/ml), Ad-GDNF (1.3 × 10^10^ PFU/ml), Ad-NCAM1 (2 × 10^10^ PFU/ml), and Ad-GFP (1.5 × 10^9^ PFU/ml) were determined by the plaque formation technique in the HEK-293 cell culture. For injection 2 × 10^7^ virus particles of Ad5-GFP in 20 μl of saline and 2 × 10^7^ virus particles mixture of Ad5-VEGF165, Ad5-GDNF, and Ad5-NCAM1 in 20 μl of saline were prepared.

### Gene Engineered UCB-MC

Human umbilical cord blood samples were obtained and processed under the license of Kazan State Medical University Stem Cell Bank. Mononuclear cells from umbilical cord blood (UCB-MC) were isolated by a standard technique of sedimentation on to a density barrier (Ficoll, 1.077 g/ml) as described previously (Islamov et al., 2015). For gene engineering, UCB-MC were seeded in 10 cm culture dishes and transduced with Ad5-GFP or simultaneously with three adenoviral vectors (Ad-VEGF165, Ad-GDNF, and Ad-NCAM1) with multiplicity of infection (MOI) of 10. Transduced cells were incubated for 12–16 h, washed in Dulbecco’s phosphate-buffered saline (DPBS, PanEco, Moscow, Russia), and prepared for xenotransplantation. The level of recombinant genes mRNA synthesis in the gene engineered UCB-MC was confirmed by RT-PCR as described earlier (Islamov et al., 2016). The reporter green fluorescent protein synthesis in the UCB-MC+Ad-GFP was observed 96 h after cell transduction using fluorescent microscopy. *In vitro* analysis of transgene expression in gene modified UCB-MC is consistent with the results documented in Islamov et al. (2016). For infusion 2 × 10^6^ UCB-MC+Ad5-GFP in 20 μl of saline and 2 × 10^6^ UCB-MC+Ad-VEGF165+Ad-GDNF+Ad-NCAM1 in 20 μl of saline were prepared.

### Animals and Treatment

Mature male Wistar rats weighing 250–300 g were obtained from Pushchino Laboratory, (Pushchino, Russia). Rats were housed one per cage under standard laboratory conditions, with a 12-h light/dark schedule, and *ad libitum* access to food and water. Brain ischemia was induced by permanent occlusion of the middle cerebral artery (MCA) (Kranz et al., 2010; Lemarchant et al., 2016). Briefly, rats (*n* = 35) were deeply anesthetized intraperitoneally with Zoletil 100 (Virbac Laboratoires, France) 3 mg/kg and Xyla (Interchemie werken “De Adelaar” B.V., Netherlands) 4.8 mg/kg. Body temperature was maintained at 37–38°Ñ with a heating pad. Through the ventral neck incision the right common carotid artery was ligated with 3.0 surgical silk. Temporal bone was separated from the skeletal muscle and a 10 mm hole was drilled in the bone. The dura was carefully removed in order to expose MCA. The occlusion of the MCA was conducted using an operating microscope by thermocoagulation. After MCA occlusion the temporal muscle was used to close the hole in the bone and the skin wound was stitched. Four hours after MCA occlusion, UCB-MC or adenoviral vectors were infused intrathecally. For intrathecal injection a laminectomy was made over the L4–L5 vertebral level. According to the injected substances the animals were divided into five experimental groups:

1. Saline (control) group: infusion of 0.9% NaCl (*n* = 5).
2. Ad-GFP (control) group: infusion of Ad5 carrying reporter gene of green fluorescent protein (*n* = 7).
3. UCB-MC+Ad-GFP (cell therapy) group: infusion of human umbilical cord blood mono-nuclear cells (UCB-MC) transduced with Ad5-GFP (*n* = 6).
4. Ad-VEGF-GDNF-NCAM (*in vivo* gene therapy) group: infusion of mixture of Ad5-VEGF165, Ad5-GDNF, and Ad5-NCAM1 (*n* = 4).
5. UCB-MC+Ad-VEGF-GDNF-NCAM (*ex vivo* gene therapy) group: infusion of human UCB-MC simultaneously transduced with Ad5-VEGF165, Ad5-GDNF, and Ad5-NCAM1 (*n* = 6).

In post-operative period, animals received antibacterial therapy for 5 days with Ceftriaxone (Sandoz, Austria) intramuscularly (50 mg/kg) once a day and analgesic therapy with Ketamin (Dr. Reddy’s Laboratories, Ltd., Hyderabad, Andhra Pradesh, India) intramuscularly (2.5 mg/kg) once a day. The animal protocol was approved by the Kazan State Medical University Animal Care and Use Committee. The mortality rate of the experimental animals during the surgery and in first post-operative week was 20% which corresponds to the severity of the surgery (Ström et al., 2013).

The ischemic damage caused by stroke develops over several hours or days. The promising period for stroke treatment is considered within 3 weeks of the injury (Wahl and Schwab, 2014). Thus for histological analysis of the gene therapy, efficacy experimental animals were sacrificed 3 weeks after MCA occlusion. Rats were deeply anesthetized by intraperitoneal injection of sodium pentobarbital (60 mg/kg) and intracardially perfused with 4% paraformaldehyde (PFA, Sigma) in phosphate-buffered saline (PBS, pH 7.4). Brains were removed and postfixed in 4% PFA at 4°C overnight and then cryoprotected in 30% sucrose, and embedded in TBS tissue freezing medium (Triangle Biomedical Science, Durham, NC, United States). Frontal sections (15 μm) were obtained through the epicenter of stroke with a cryostat (Microm HM 560, Thermo Scientific, Waltham, MA, United States).

### Evaluation of the Cerebral Infarction

To determine the volume of the cerebral infarction digital images were evaluated from every 10 frontal brain sections (with 200 μm interval) through the stroke area after staining with hematoxylin and eosin. For calculation of the volume (*V*), the depth (*h*) and diameter (2 × *r*) of the infarct from each digital image were determined using ImageJ software. The calculation was carried out using the formula: *V* = 1/3 × *h*_max_ × π × *r*_max_^2^, where *h*_max_ is the maximum depth and the *r*_max_ the maximum radius.

### Immunofluorescent Staining

Frontal brain 20 μm sections were incubated with primary antibodies (Ab) overnight at 4°C. Primary antibodies (Ab) reacting with cell-specific markers: HuNA for UCB-MC, glial fibrillary acidic protein (GFAP) for astrocytes, Olig2 for oligodendrocytes, and Iba1 for microglial cells and primary Abs against recombinant proteins (VEGF, GDNF, and NCAM), synaptic proteins (PSD95 and *synaptophysin*), heat shock proteins (Hsp70), and pro-apoptotic proteins (Caspase 3) were applied (**Table 1**).

**Table 1:** Primary and secondary antibodies used in immunofluorescent staining.

Antibody	Host	Dilution	Source
GDNF	Rabbit	1:100	Santa Cruz
VEGF	Goat	1:300	Sigma
GFAP	Mouse	1:200	Santa Cruz
Iba1	Rabbit	1:150	Biocare Medical
HNA	Mouse	1:150	Millipore
HSP70	Rabbit	1:200	Abcam
Olig2	Rabbit	1:100	Santa Cruz
NCAM	Rabbit	1:100	Santa Cruz
PSD95	Rabbit	1:200	Abcam
Synaptophysin conjugated with Alexa 488	Rabbit	1:100	Abcam
Caspase 3	Rabbit	1:200	Abcam
Anti-rabbit IgG conjugated with	Donkey	1:200	Invitrogen
Alexa 647			
Anti-mouse IgG conjugated with	Donkey	1:200	Invitrogen
Alexa 655			

Secondary Alexa Fluor 647 and 555 conjugated IgG (Invitrogen) were applied in 1:200 dilution for 1.5 h at room temperature. Sections were then stained with DAPI (Sigma) or PI (Sigma) for 12 min and embedded in glycerol (GalenoPharm, Saint Petersburg, Russia) (Niu et al., 2015). Digital images were captured using a LSM 510-Meta Microscope (Carl Zeiss, Oberkochen, Germany). All sections were imaged in the *z*-stack by using identical confocal settings (laser intensity, gain, offset) and for each channel the lowest intensity signals within a *z*-stack were removed to minimize background. Digital images at the site of the stroke were obtained from 10 adjacent optical slices of *z*-stack using a ×40 objective lens.

The numbers of immunopositive cells for GFAP, Olig2, Iba1, and Caspase 3 were quantified in area 0.05 mm^2^. The cells with nuclei clearly visualized by DAPI were evaluated. The levels of synaptophysin, PSD95, and Hsp70 immunoexpression were assessed according to the fluorescence density of corresponding markers in area 0.05 mm^2^. All images were analyzed following the same semi-automated in-house algorithm. The arithmetic mean intensity of the fluorescence density value was obtained using the software Zen 2012 (Carl Zeiss, Oberkochen, Germany) and expressed as mean intensity fluorescence units. In double immunofluorescent staining, the genuine co-localization of HuNA and one of the recombinant molecules (VEGF, GDNF, and NCAM) was confirmed by analyzing three channel merged images. Sections incubated without any Ab or with secondary Ab alone were analyzed to ensure visualization of specific Ab applied. Investigated sections were validated by two researchers blind to the tissues presented and in order to ensure the correct identification of immunoreactivity patterns.

### Statistical Analysis

To determine statistical significance of the morphometric and immunofluorescent staining data between experimental groups, which included different numbers of animals, we used the Kruskal–Wallis test as nonparametric method for testing whether samples originate from the same distribution. When a null hypothesis was rejected, we used Dunn’s test as a *post hoc* method for comparison of each therapeutic group with each control group. R language version 3.3.1 (R Foundation for Statistical Computing, Vienna, Austria) was employed. Samples were visualized using box plots, descriptive statistics are presented as median (minimum; maximum). Non-adjusted *p* < 0.05 was considered statistically significant.

## Results

### Cerebral Infarction Volume Analysis

**FIGURE 1:**
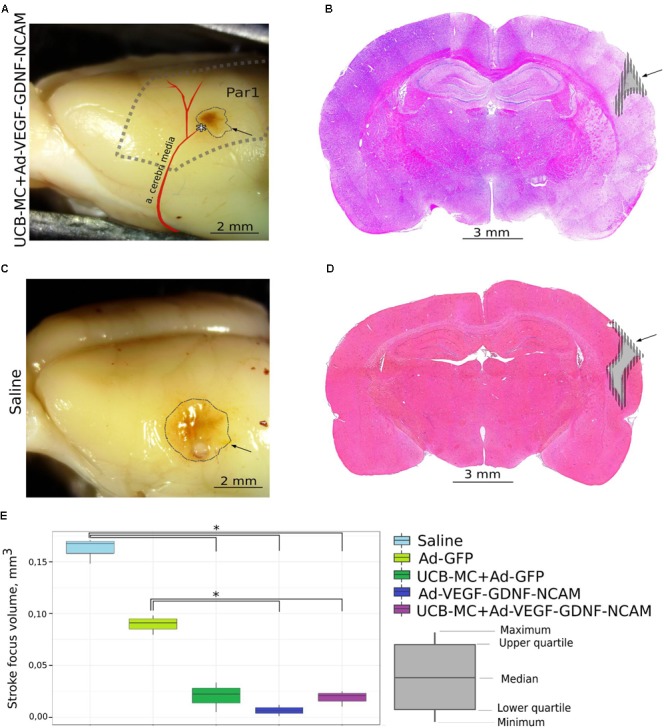
Ischemic stroke 21 days after the distal permanent occlusion of the middle cerebral artery (MCA) in rats from control (Saline) and *ex vivo* (UCB-MC+AdVEGF-GDNF-NCAM) gene-treated groups. **(A,C)** The brain cortex infarction area. ^∗^The site of the MCA occlusion. Par1, cortex parietals, area 1. **(B,D)** Frontal frozen section through the stroke area stained with hematoxylin and eosin. Arrow points to the brain cortex infarction in the left hemisphere. **(E)** Morphometric analysis of cerebral infarction volume. Data are visualized using box plots. ^∗^Difference between experimental groups, *p* < 0.05.

Morphological evaluation of rat brain cortex 3 weeks after surgery revealed an infarct zone located in the parietal lobe [parietal cortex, area 1 (Par1)] which corresponds to the site of MCA occlusion (**Figure 1**). Morphometric analysis of the infarct volume showed the differences between therapeutic and control groups. The volume of the brain cortex infarct was significantly less in Ad-VEGF-GDNF-NCAM (0.007 [0.001; 0.012]) and UCB-MC+Ad-VEGF-GDNF-NCAM (0.021 [0.010; 0.025]) groups, when compared with control saline (0.168 [0.148; 0.171]) and Ad-GFP (0.091 [0.080; 0.098]) groups. In the UCB-MC+Ad-GFP (0.023 [0.005; 0.033]) group, the infarction volume did not differ from gene-treated groups and was lower when compared with the saline group, *p* < 0.05.

### Molecular and Cellular Changes in Brain Cortex Induced by *in Vivo* and *ex Vivo* Triple Gene Therapy at the Side of MCA Occlusion

#### Hsp70

**FIGURE 2:**
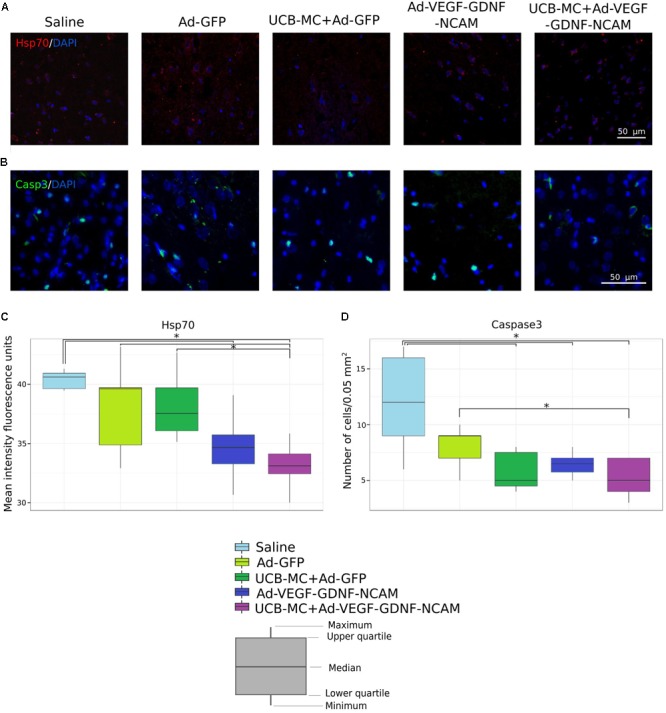
Immunoexpression of Hsp70 and Caspase 3 in brain cortex 21 days after the distal permanent occlusion of the MCA. **(A,B)** Immunofluorescent staining with Abs against Hsp70 **(A)** and Caspase 3 **(B)**. Nuclei (blue) were counterstained with DAPI. Scale bar = 50 μm. **(C)** Fluorescence density value of Hsp70 immunoexpression level. **(D)** Number of Caspase 3 immunopositive cells. Data are visualized using box plots. ^∗^Difference between experimental groups, *p* < 0.05.

Injured neurons noticeably increase expression of heat shock proteins. We measured Hsp70 levels by immunofluorescent staining (**Figures 2A,C**). The highest level of Hsp70 was documented in saline control (40.6 [39.5; 41.3]) and Ad-GFP (39.6 [32.9; 43.2]) groups. The level of the Hsp70 immunoexpression in gene-treated Ad-VEGF-GDNF-NCAM (34.7 [30.7; 39.1]) and UCB-MC+Ad-VEGF-GDNF-NCAM (33.1 [30.0; 35.8]) groups was significantly downregulated when compared with saline control group. In cell-treated UCB-MC+Ad-GFP (38 [35.1; 42.7]) group, the level of the Hsp70 did not differ from control groups and was significantly higher than in UCB-MC+Ad-VEGF-GDNF-NCAM group.

#### Caspase 3

Apoptosis of brain neurons continues to develop over several days in the peri-infarct zone (penumbra). Immunofluorescent staining with Abs against the pro-apoptotic protein Caspase 3 was used to estimate the degree of apoptosis by counting the number of Caspase 3 immunopositive cells in the stroke area. The significant decrease of Caspase 3 positive cells was revealed in all therapeutic groups in comparison with saline control (12 [6; 17]) group (**Figures 2B,D**). Amount of Caspase 3 positive cells in therapeutic UCB-MC+Ad-VEGF-GDNF-NCAM (5 [3; 7]), Ad-VEGF-GDNF-NCAM (7 [5; 8]), and UCB-MC+Ad-GFP (5 [4; 8]) groups did not differ. The number of Caspase 3 positive cells in Ad-GFF (9 [5; 10]) did not differ from Saline group and was significantly higher in comparison with UCB-MC+Ad-VEGF-GDNF-NCAM group.

#### Synaptophysin

**FIGURE 3:**
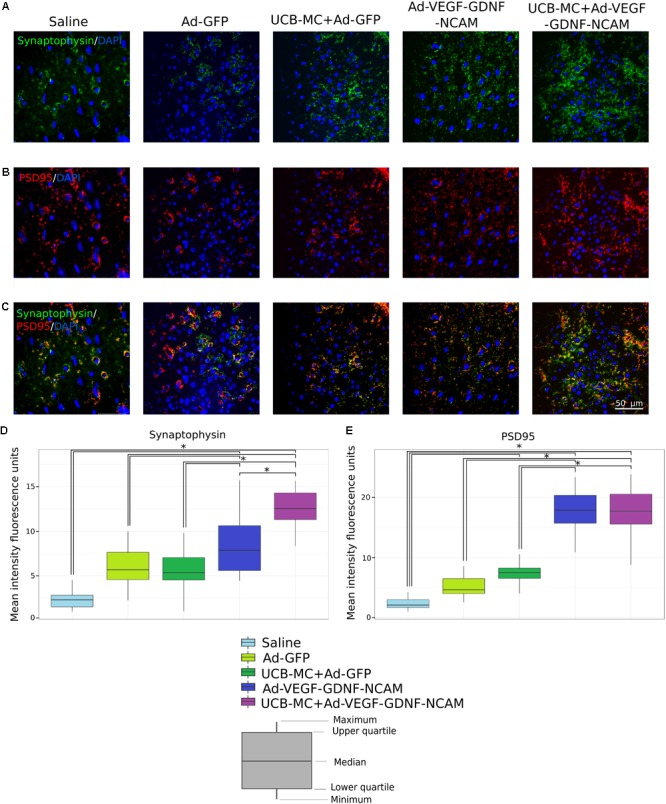
Immunoexpression of synaptic proteins in brain cortex 21 days after the distal permanent occlusion of the MCA. **(A,B)** Immunofluorescent staining with Abs against synaptophysin **(A)** and PSD95 **(B)**. **(C)** Merged image of PSD95 **(A)** and synaptophysin **(B)** immunoexpression. Nuclei (blue) were counterstained with DAPI. Scale bar = 50 μm. **(D,E)** Fluorescence density value of synaptophysin **(D)** and PSD95 **(E)** immunoexpression. Data are visualized using box plots. ^∗^Difference between experimental groups, *p* < 0.05.

The level of synaptophysin in UCB-MC+Ad-VEGF-GDNF-NCAM group (12.6 [8.3; 18.3]) was significantly higher in comparison with all experimental groups (**Figures 3A,C,D**). The immunoexpression of synaptophysin in Ad-VEGF-GDNF-NCAM (7.9 [4.5; 16.1]) group was higher when compared with UCB-MC+Ad-GFP (5.4 [1.0; 9.8]) and control groups. The level of synaptophysin in UCB-MC+Ad-GFP group did not differ from saline control (2.3 [1.0; 4.5]) and Ad-GFP (5.7 [2.3; 10.0]) groups.

#### PSD95

The mean fluorescence intensity of the PSD95 in gene-treated Ad-VEGF-GDNF-NCAM (17.9 [10.9; 26.0]) and UCB-MC+Ad-VEGF-GDNF-NCAM (17.7 [8.8; 26.7]) groups was significantly higher when compared with UCB-MC+Ad-GFP and control groups (**Figures 3B,C,E**). Immunoexpression of PSD95 in UCB-MC+Ad-GFP (7.5 [4.0; 10.5]) group did not differ from Ad-GFP groups (4.7 [2.6; 8.6]) but was higher than in saline (2.1 [1.0; 4.2]) group.

#### GFAP

**FIGURE 4:**
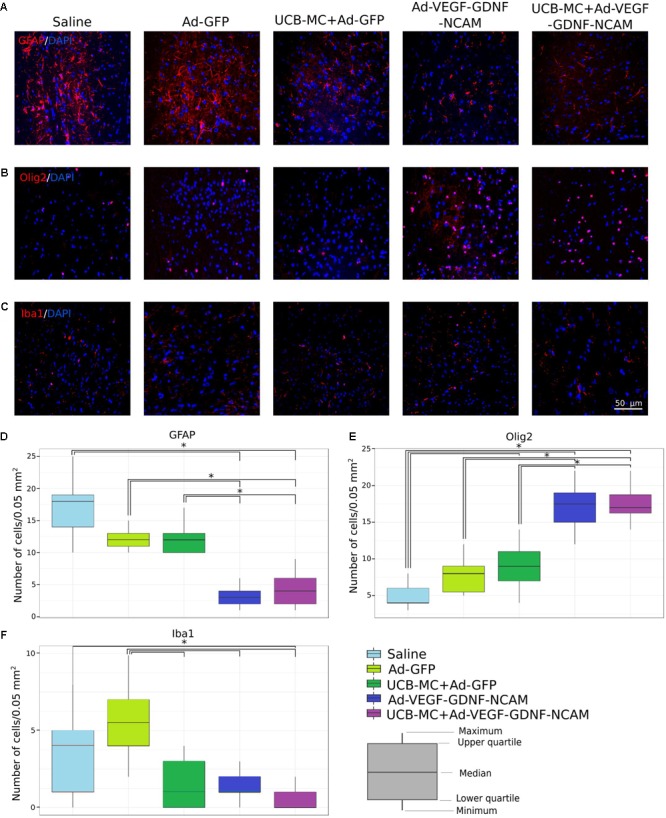
Reaction of glial cells in brain cortex on triple gene therapy 21 days after the distal permanent occlusion of the MCA. **(A–C)** Immunofluorescent staining of astrocytes with Abs against GFAP **(A)**, oligodendroglial cells with Abs against Olig2 **(B)**, and microglial cells with Abs against Iba1 **(C)**. Nuclei (blue) were counterstained with DAPI. Scale bar = 50 μm. **(D–F)** Number of GFAP immunopositive cells **(D)**, number of Olig2 immunopositive cells **(E)**, and number of Iba1 immunopositive cells **(F)**. Data are visualized using box plots. ^∗^Difference between experimental groups, *p* < 0.05.

In brain cortex, GFAP is expressed exclusively in astrocytes which play an important role in response to CNS disease. In the present study, we counted the number of GFAP-positive astrocytes in stroke area. The morphometric analysis showed significantly reduced amount of the GFAP-positive astrocytes in gene-treated Ad-VEGF-GDNF-NCAM (3 [1; 6]) and UCB-MC+Ad-VEGF-GDNF-NCAM (4 [1; 9]) groups when compared with saline control (18 [10; 25]) and Ad-GFP (12 [10; 15]) groups (**Figures 4A,D**). Number of GFAP-positive cells in UCB-MC+Ad-GFP (12 [10; 17]) group was higher than in gene-treated groups and did not differ from control groups.

#### Olig2

Oligodendrocytes responsible for production and maintaining myelin in CNS are vulnerable to damage and death in a variety of neurological disorders. In immunofluorescent staining, we used Olig2 as a marker to quantify **oligodendroglial progenitor and mature cells**. The number of **oligodendroglial cells** was significantly higher in gene-treated Ad-VEGF-GDNF-NCAM (18 [12; 22]) and in UCB-MC+Ad-VEGF-GDNF-NCAM (17 [14; 24]) groups in comparison to saline control (4 [3; 8]) and Ad-GFP (8 [5; 12]) groups and UCB-MC+Ad-GFP (9 [4; 14]) group (**Figures 4B,E**). The quantity of Olig2-positive cells did not differ from Ad-GFP group but was significantly higher when compared to saline controls.

#### Iba1

Microglia-/macrophage-specific calcium-binding adaptor molecule 1 (Iba1) was used as a marker for quantifying both quiescent and reactive microglial cells. In the ischemic core and peri-infarct zone, microglial cells secrete proinflammatory cytokines and result in ischemic damage (Taylor and Sansing, 2013). In our study, the numbers of Iba1-immunopositive cells in all UCB-MC+Ad-GFP (1 [0; 4]), Ad-VEGF-GDNF-NCAM (1 [0; 3]), and UCB-MC+Ad-VEGF-GDNF-NCAM (0 [0; 2]) therapeutic groups did not differ and were significantly lower in comparison with control Ad-GFP group (6 [2; 10]) (**Figures 4C,F**). Notably, in saline controls (4 [0; 8]), the number of microglia cells was significantly higher than in UCB-MC+Ad-VEGF-GDNF-NCAM group.

### Immunoexpression of the Reporter and Therapeutic Transgenes after Viral- and Cell-Mediated Intrathecal Gene Delivery

**FIGURE 5:**
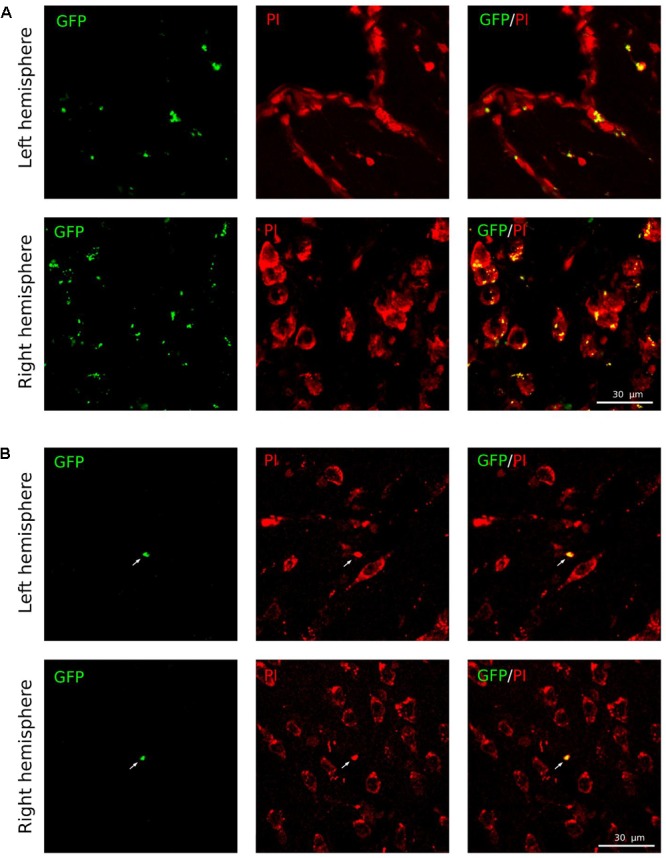
Expression of the reporter GFP gene in brain cortex 21 days after intrathecal infusion of Ad-GFP or UCB-MC+Ad-GFP. **(A)** Transduction of brain cortex cells with Ad-GFP in the ischemic left and right hemispheres. **(B)** Expression of GFP in UCB-MC *ex vivo* transduced with Ad-GFP in the ischemic left and right hemispheres. Green, GFP; red, propidium iodide (PI). Scale bar = 30 μm.

The expression of the reporter GFP gene was studied on 21 days after intrathecal injection of Ad-GFP or UCB-MC transduced with Ad-GFP. As expected, cerebrospinal fluid (CSF) flow facilitated dissemination of the viral vectors and UCB-MC+Ad-GFP throughout the CNS. In a previous study, we demonstrated expression of reporter GFP gene along the spinal cord following intrathecal injection of Ad-GFP (Izmailov et al., 2017). In the present study, the specific fluorescence of GFP in brain cells was visualized at the site of stroke and in the opposite hemisphere after viral-mediated GFP gene delivery (**Figure 5A**). In the cell-mediated approach of gene delivery the reporter GFP expression was restricted to transplanted genetically modified UCB-MC (**Figure 5B**).

**FIGURE 6:**
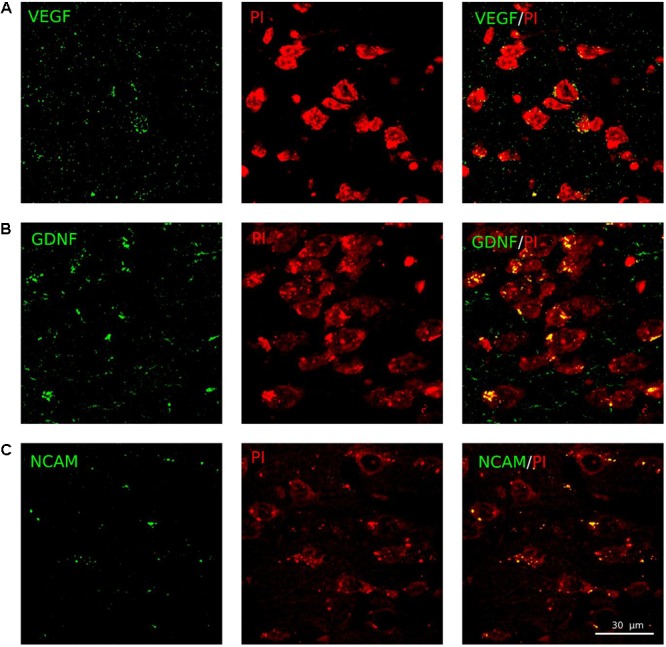
Ad5-VEGF, Ad5-GDNF, and Ad5-NCAM transduction into the brain cortex cells. Immunofluorescent staining (green fluorescence) of the recombinant VEGF **(A)**, GDNF **(B)**, and NCAM **(C)** at 21 days after intrathecal injection of mixture of three adenoviral vectors carrying corresponding genes. Brain cortex cells were counterstained with PI. Scale bar = 30 μm.

The expression of recombinant therapeutic genes encoding VEGF, GDNF, and NCAM in brain cortex was investigated 3 weeks after simultaneous intrathecal injection of three adenoviral vectors (Ad-VEGF+Ad-GDNF+Ad-NCAM). Expression of the therapeutic genes was documented using specific Ab against the target molecules. Immunofluorescent analysis revealed expression of VEGF, GDNF, and NCAM in neural and glial cells at the site of stroke (**Figure 6**). Notably, the data on the level of the reporter and therapeutic genes expression correspond to the short period of expression for adenoviral vectors and are consistent with previously described results (Izmailov et al., 2017).

**FIGURE 7:**
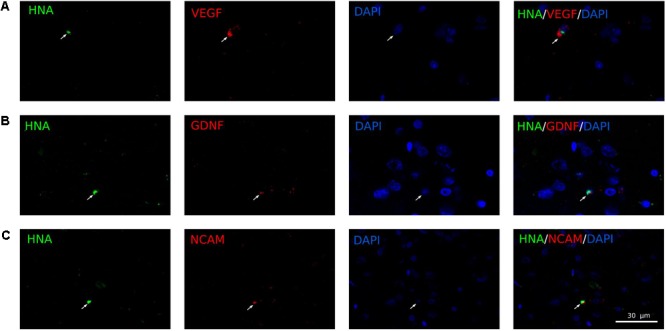
Double immunofluorescent staining of UCB-MC in brain cortex 21 days after intrathecal infusion. Anti-human nuclear antigen (HNA) antibodies (Ab) were used for identification of UCB-MC, recombinant human proteins were identified with Ab against human specific VEGF, GDNF, and NCAM. **(A)** HNA immunopositive cells (green) expressing VEGF (red). **(B)** HNA immunopositive cells (green) expressing GDNF (red). **(C)** HNA immunopositive cells (green) expressing NCAM (red). Morphology of HNA immunopositive cells corresponds to the cord blood mononuclear cells with round nuclei surrounded by a thin rim of cytoplasm. Scale bar = 30 μm.

The expression of the therapeutic genes in the transplanted genetically modified UCB-MC was investigated using a double immunofluorescent staining. Anti-human nuclear antigen (HNA) Ab were used for identification of UCB-MC in brain cortex and the specific Abs to VEGF, GDNF, and NCAM were applied to confirm efficacy of recombinant gene expression in UCB-MC *in vivo*. Three weeks after intrathecal injection of gene modified UCB-MC the HNA-positive cells were found at the site of stroke. The double staining of HNA and markers of target genes revealed that gene modified UCB-MC are able to produce therapeutic molecules (Ad-VEGF+Ad-GDNF+Ad-NCAM) in brain cortex up to 21 days after intrathecal transplantation (**Figure 7**).

## Discussion

Previously we used the approach of *ex vivo* gene therapy for amyotrophic lateral sclerosis (Islamov et al., 2015, 2016) and SCI (Chen et al., 2014; Islamov et al., 2017a). Based on the data we proposed that UCB-MC transduced with adenoviral vectors carrying genes encoding growth and neurotrophic factors can rescue spinal neurons from cell death in those diseases. Non-integrating adenoviral vectors are widely used for *in vivo* (direct) gene therapy in experiments and in patients for the production of biologically active molecules in diseases of the CNS such as stroke, neurotrauma, and neurodegeneration (Lim et al., 2010). Recombinant replication-defective adenoviruses are known to accommodate large recombinant genes (up to 8 kB) and have a high level of non-mitotic cell transduction. In the infected cells, adenoviral vectors may hold expression of therapeutic genes for about a month (Abdellatif et al., 2006). The negative issue of the adenoviral vectors usage for direct gene therapy is their high immunogenicity. *Ex vivo* gene therapy may overcome this problem by reducing the response of the recipient immune system against the viral antigens. Mesenchymal cells engineered to produce VEGF (Chen et al., 2015), BDNF (Nomura et al., 2005), and placental growth factor (Liu et al., 2006) were used for stroke treatment in a rat model. In the past decade, transfusion of UCB-MC was suggested for neural repairs (Sun and Ma, 2013). UCB-MC as therapy for non-hematopoietic disorders does not need HLA matching since the proposed mechanism of the therapeutic action of UCB-MC is based on their production of biologically active molecules (cytokines, chemokines, growth, and neurotrophic factors). To enhance the efficacy of UCB-MC and make cell therapy more specific the use of gene engineered UCB-MC carrying therapeutic genes aimed to produce particular molecules to battle the disease is under intensive study. The rationale for employing UCB-MC to deliver therapeutic genes has been proposed for the treatment of ischemic cardiovascular diseases (Ikeda et al., 2004; Chen et al., 2005).

Thus the strategy to use gene engineered UCB-MC over expressing neurotrophic factors is a promising method for *ex vivo* or cell-mediated gene therapy for the treatment of neurological disorders. Currently, gene therapy for stroke is being actively studied in animal models (Yamashita et al., 2009; Craig and Housley, 2016). However, for now, there are no approved and effective drugs containing genetic material to treat stroke patients. In the present paper, we performed the comparative study of (1) direct (*in vivo*) gene therapy, (2) cell therapy, and (3) cell-mediated (*ex vivo*) gene therapy for stroke in a rat model.

### Direct (*in Vivo*) Gene Therapy

The list of the genes encoding potential neuroprotective molecules is large and selection of the therapeutic molecules or their combinations is based on the research need. In our studies we employed genes encoding VEGF, GDNF, and NCAM. VEGF has specific mitogenic activity against endothelial cells (Ferrara et al., 1992). However, in addition to its angiogenic action, VEGF also demonstrates the properties of a typical neurotrophic factor. It supports the survival of sensory (Sondell et al., 1999) and motor (Islamov et al., 2004) neurons, stimulates proliferation of neural stem cells (Jin et al., 1999), astrocytes (Rosenstein et al., 1998), as well as Schwann cells (Sondell et al., 1999). GDNF has a significant neuroprotective effect on the neurons of the brain and spinal cord (Cheng et al., 2002) and stimulates growth of nerve processes (Iannotti et al., 2003). NCAM is expressed on the surface of neurons and neuroglia. NCAM-mediated intercellular interactions ensure survival and migration of neurons, directed growth of neurites, and synaptogenesis in a course of neuronal ontogenesis and regeneration. For gene delivery the local intracerebral or intraventricular injections of viral vectors carrying various therapeutic genes is widely used. In the present study, we demonstrated that intrathecal injection of three Ad5 carrying genes encoding VEGF, GDNF, and NCAM 4 h following distal occlusion of MCA in rats had a significant effect on the sparing of ischemic brain cortex and recovery of the neural and glial cells in the insult area. After intrathecal injection adenoviral vectors easily spread and transduce the neural and glial cells throughout CNS via CSF. The expression of recombinant genes was demonstrated 3 weeks after delivery at site of ischemic stroke (**Figure 7**). Recombinant molecules VEGF, GDNF, and NCAM exerted their neurotrophic action on neurons in penumbra increasing their survivability. The role of VEGF in stroke treatment may be beneficial for angiogenesis as well. Recovery of microvascular circulation modulates the function of glial cells which in turn positively affects the neural cells.

### Cell Therapy

Stem cells of different origin have been studied in experiments for ischemic stroke cell therapy. A positive effect in rats with ischemic stroke was established after intravenous (Boltze et al., 2012) or intra-arterial (Karlupia et al., 2014) xenotransplantation of UCB-MC. The reduction in neurologic deficit and brain infarction area was significantly higher after intra-arterial infusion of UCB-MC when compared with the MSC obtained from umbilical cord. In clinical trials, autologous cells isolated from bone marrow (Jeong et al., 2014) or peripheral blood were preferentially used for post stroke transplantation (Wang et al., 2013; Chernykh et al., 2016). Based on our previous studies we confirmed the rationale for using UCB-MC in the treatment of neurological disorders such as amyotrophic lateral sclerosis and SCI (Islamov et al., 2016, 2017a). The reason for clinical application of UCB-MC is their suitability for human allo- and autotransplantation, as well as low immunogenicity, availability, ease of preparation, and storage (Yang et al., 2010). An important factor is the absence of legislative, ethical, or religious prohibitions related to the transplantation of human umbilical cord blood cells. In addition, mononuclear cells of the umbilical cord contain different stem cells that can act as a source of numerous growth and trophic factors, being able to give rise to specialized cells of different tissues and stimulate angiogenesis (Ballen et al., 2013). Here, we showed that UCB-MC after intrathecal infusion in a rat stroke model migrate to the site of brain infarct, survive for 3 weeks after transplantation, and preserve brain cortex from degeneration. UCB-MC have their own endogenous potential to increase survivability of the affected neural and glial cells in the penumbra. The results are consistent with our previous data demonstrating the positive effect of UCB-MC on regeneration of spinal cord after severe contusion (Izmailov et al., 2017). Based on these results and on the efficacy of direct triple gene therapy for stroke we proposed to enhance the therapeutic potential of UCB-MC via simultaneous transduction of UCB-MC with Ad-VEGF, Ad-GDNF, and Ad-NCAM.

### Cell-Mediated (*ex Vivo*) Gene Therapy

The current study is designed as a proof-of-concept *in vivo* study of the therapeutic efficacy of UCB-MC-mediated triple gene therapy for stroke. Intrathecal transplantation of the gene–cell construct (UCB-MC+Ad-VEGF+GDNF+NCAM) 4 h after distal occlusion of MCA in rat revealed gene modified cells in the stroke area 3 weeks after transplantation. The cells produced recombinant molecules VEGF, GDNF, and NCAM which are thought to intensify the endogenous neuroprotective effect of UCB-MC on ischemic brain cells. The random distribution of the UCB-MC along the spinal cord and brain after intrathecal transplantation may result in the limited number of UCB-MC observed in the stroke area (**Figures 5, 6**). Moreover, some of the transplanted UCB-MC may stay circulating in the CSF and, as host leukocytes, may migrate from the CSF into bloodstream across the choroid plexus. To increase the targeting of UCB-MC into the nervous tissue we used Ad5-NCAM where NCAM is thought to be responsible for targeting, migration, and viability of the transplanted UCB-MC. In our previous research we documented this targeting of UCB-MC in lumbar spinal cord after intravenous (Safiullov et al., 2015) and intrathecal injection (Izmailov et al., 2017). Meanwhile, localization of the gene modified UCB-MC does not diminish their therapeutic efficacy since the biologically active recombinant molecules can be easily circulated by CSF to affected areas.

### Comparison of Gene, Cell, and Gene–Cell Therapy

The comparative analysis of direct gene therapy, cell therapy, and cell-mediated gene therapy for stroke in rat model revealed the superiority of triple gene (VEGF+GDNF+NCAM) therapy over UCB-MC therapy. In addition, the infarct volume and the number of Caspase 3-positive cells did not differ in UCB-MC-treated and gene-treated animals. The most prominent positive effect of the therapeutic genes was observed in the response of neural and glial cells in the stroke area. Thus in the gene-treated animals, the decrease of Hsp70 level and higher expression of synaptic proteins (PSD95 and synaptophysin) suggest the therapeutic efficacy of recombinant molecules (VEGF+GDNF+NCAM) on survivability and synaptic function recovery of neural cells in the penumbra. These results are also in line with data demonstrating reduced astrocytosis (decreased number of GFAP-positive astrocytes and Iba1-positive microglial cells) and maintained myelination (increased number of Olig2-positive cells) in rats receiving *in vivo* or *ex vivo* gene therapy. Comparison of *in vivo* and *ex vivo* gene therapies demonstrated some advantages of UCB-MC-mediated gene therapy. Synergistic effect of UCB-MC and therapeutic genes was observed in the lowering of Hsp70 level and increasing of synaptophysin expression. These results are consistent with our previous study which revealed that the UCB-MC+Ad-VEGF+GDNF+NCAM gene–cell construct had a positive effect on functional recovery and the molecular and cellular changes leading to post-traumatic spinal cord regeneration (Izmailov et al., 2017).

## Conclusion

Triple gene therapy demonstrated the positive effect on sparing and morpho-functional recovery of brain cortex in stroke area. The efficacy of the *ex vivo* gene therapy (intrathecal transplantation of UCB-MC transduced with Ad5 carrying VEGF, GDNF, and NCAM genes) showed some advantages over the *in vivo* gene therapy (intrathecal injection of three Ad5 carrying therapeutic genes). Thus, based on our previous investigations and the present study we propose a strategy of using genetically engineered human UCB-MC as carriers for the therapeutic genes encoding neurotrophic factors to treat neurodegenerative diseases such as ALS, SCI, and stroke. However, further investigations on large animals with similar anatomy and physiological characteristics to man are required. It is highly important in pre-clinical studies to solve the potential clinical obstacles such as immunogenicity of the UCB-MC, the level of the produced recombinant therapeutic molecules and their possible side effects.

## Ethics Statement

The experimental protocol was consistent with the recommendations of the Physiological Section of the Russian National Committee on Bioethics. All animal treatments, anesthesia, surgery, post-operative care, perfusion, and euthanasia at the endpoints were approved by the Kazan State Medical University Animal Care Committee (Permit Number 6, from 20 June 2017). All procedures with human umbilical cord blood cells were performed according to license FS-16-01-001421 (13 April 2016).

## Author Contributions

RI, AP, and BN designed the model framework and wrote the manuscript. MES, FB, VM, TP, FF, AI, MK, ZS, and MMS conducted the experiments, collected and analyzed the data, and contributed to the manuscript writing.

## Conflict of Interest Statement

The authors declare that the research was conducted in the absence of any commercial or financial relationships that could be construed as a potential conflict of interest.
